# Comparison of different volumes spread of erector spinae block in postmastectomy pain syndrome management: a prospective randomized comparative study

**DOI:** 10.1186/s12871-023-02239-1

**Published:** 2023-08-22

**Authors:** Nevert A. Abdelghaffar, Ghada F. Amer

**Affiliations:** Department of Anesthesia, Surgical Intensive Care and Pain Management, Faculty of Medicine, Mansoura University, PO: 35516, Mansoura, Egypt

**Keywords:** Different volumes, Erector spinae, Postmastectomy pain

## Abstract

**Background:**

Postmastectomy pain is chronic pain that occurs in females after breast surgeries. In this study, we estimated the vertebral levels reached by two different volumes (20 ml and 30 ml solutions) in the erector spinae block (ESB), as well as assess pain improvement and patient satisfaction in females with postmastectomy pain syndrome.

**Methods:**

Fifty patients were assigned to two groups. The 20 ml group received ESB with 10 ml of bupivacaine 0.5%, 1 ml of 40 mg/ml of methylprednisolone, 2 ml of non-ionic contrast, and 7 ml of saline 0.9%. The 30 ml group received ESB with 15 ml of bupivacaine 0.5%, 1 ml of 40 mg/ml of methylprednisolone, 2 ml of non-ionic contrast, and 12 ml of saline 0.9%.

**Results:**

The mean numbers of the blockade segments were 5.12 ± 0.726 and 6.36 ± 0.569 in the 20 ml and 30 ml groups, respectively (*P* < 0.001). The T1 to T6 blockade levels were achieved in six patients (24%) in the 20 ml group, versus 23 patients (92%) in the 30 ml group (*P* < 0.001). The numerical rating scale (NRS) improved in the 30 ml group during the follow-up period, compared to the 20 ml group. The T1 to T6 blockade levels showed better NRS (*P* < 0.001) and patient satisfaction (*P* = 0.011) than other blockade levels.

**Conclusions:**

The injection of a 30 ml solution of 0.25% bupivacaine with methylprednisolone in erector spinae block (ESB) may result in better analgesia and higher patient satisfaction in individuals with postmastectomy pain syndrome (PMPS) compared to a 20 ml solution.

**Trial registration:**

ClinicalTrials.gov (NCT05192278) on 14/1/2022.

## Background

Breast cancer is the most prevalent cancer among women. Postmastectomy pain syndrome (PMPS) is one of the most common postoperative complications that requires effective and safe management [1]. It is a neuropathic pain that occurs after quadrantectomy or mastectomy surgeries and persists for more than 3 or 6 months postoperatively [2]. PMPS is characterized by pain in the anterior aspect of the chest wall, axilla, and upper medial arm, which can lead to frozen shoulder or complex regional pain syndrome (causalgia). The pain may manifest as burning, stinging, stabbing, shooting, tingling, or hyperesthesia [3]. Managing PMPS is often challenging and can be achieved through various methods such as multimodal analgesia and regional techniques [4, 5].

Erector spinae block (ESB) is a relatively new technique used to manage acute postoperative pain in different surgeries, as well as chronic pain syndromes. Although its mechanism is not fully understood and most publications consist of case reports and personal clinical experiences, the available data agree on its simplicity and safety compared to bilateral paravertebral blocks or thoracic epidural blocks [6, 7].

ESB can be performed using either the superficial technique, located between the large rhomboid muscles and the erector spinae muscle, or the deep technique, beneath the erector spinae muscle [8]. Recently, ESB has been widely used for various neuropathic pain conditions. However, previous studies have not reached a consensus on the optimal volume for each condition, with total volumes ranging from 10 to 30 ml [5, 9].

In this study, we aimed to determine the vertebral levels reached by two different volumes (20 ml and 30 ml solutions) in the erector spinae block (ESB), as well as assess pain improvement and patient satisfaction in females with postmastectomy pain syndrome.

## Methods

### Study design and participants

We conducted a prospective randomized comparative pilot study in the pain clinics of Mansoura University Hospital, Egypt, from January 2022 to September 2022. We obtained approval from the Institutional Research Board (IRB) of the Mansoura University Faculty of Medicine on 28/11/2021 (Code number: R.21.11.1523.R1). The study was registered on Clinicaltrials.gov on 14/1/2022 (NCT05192278) and was conducted in accordance with the Helsinki Declaration. After providing a clear explanation of the study details, we obtained informed written consent from all study participants.

The inclusion criteria were as follows: females who had undergone mastectomy surgery more than six months ago, aged over 18 years old, experiencing postmastectomy pain that was not relieved by medical treatments alone (such as magnesium 100 mg/day, anticonvulsants, antidepressants, or EMLA cream), with a Numeric Rating Scale (NRS) score greater than four. Additionally, participants needed to have a body mass index (BMI) of less than 30 and be classified as American Society of Anesthesiology Physical Status (ASAPS) class I or II. The NRS was used to assess and express their pain levels, ranging from 0 to 10, with ten representing extreme pain and zero representing no pain.

The exclusion criteria were as follows: patient rejection, systemic or local sepsis, allergy to the medications used in the study, coagulopathy, distorted local anatomy, uncontrolled cardiovascular diseases, neurological deficits, respiratory disorders, psychiatric disorders, or a history of drug abuse.

### Sample size calculation

We used the Power Analysis and Sample Size (PASS) software program, version 15.0.5 for Windows 2017, to calculate the sample size. Primary data were obtained from a pilot study conducted in Mansoura University Hospital, which included 12 patients. The primary outcome was the number of vertebral levels reached by the dye. Participants were assigned to either the 20 ml group or the 30 ml group. The number of levels reached was 5.6 ± 1.02 for the 20 ml group and 6.8 ± 1.17 for the 30 ml group, with a difference of 1.2 levels. To achieve 95% power in the proposed study, using a two-sided two-sample unequal-variance t-test with a significance level of 5%, a sample size of 23 patients in each group was required. Assuming a dropout rate of 10%, the number of patients in each group was set at 25.

### Randomization (Fig. 1)

**Fig. 1:**
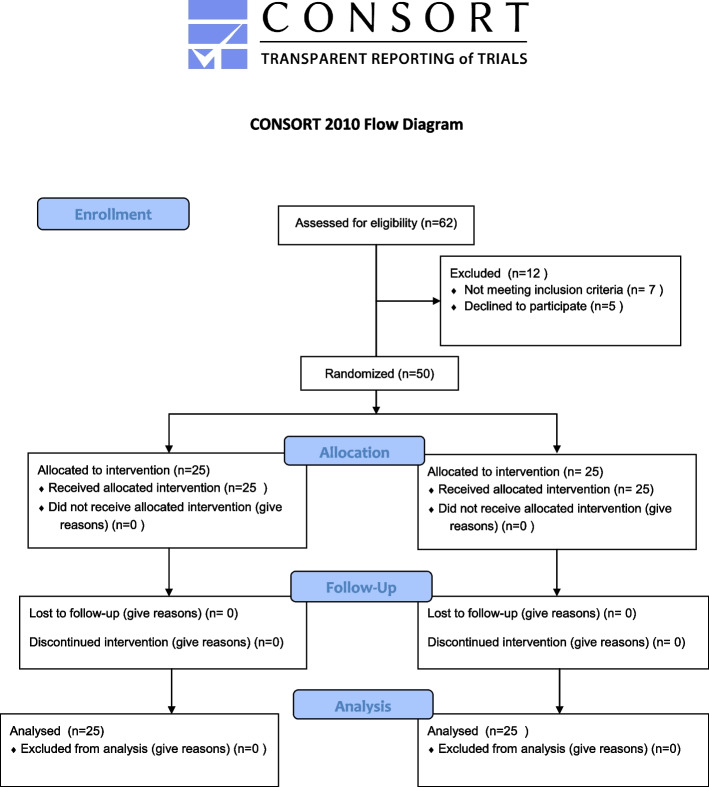
Consort flow chart

Randomization was performed using a computer-generated list of random numbers in a 1:1 ratio. The distribution results were kept in an opaque envelope by the study administrator. On the day of the block, the study manager provided the envelope to the anesthesiologist who performed the block.

### Grouping

- The 20 ml group (*n* = 25) received ESB with 10 ml of bupivacaine 0.5%, 1 ml of 40 mg/ml of methylprednisolone, 2 ml of non-ionic contrast, and 7 ml of saline 0.9% (with a total volume of 20 ml of bupivacaine 0.25%).
- The 30 ml group (*n* = 25) received ESB with 15 ml of bupivacaine 0.5%, 1 ml of 40 mg/ml of methylprednisolone, 2 ml of non-ionic contrast, and 12 ml of saline 0.9% (with a total volume of 30 ml of bupivacaine 0.25%).

### ESB technique

**Fig. 2:**
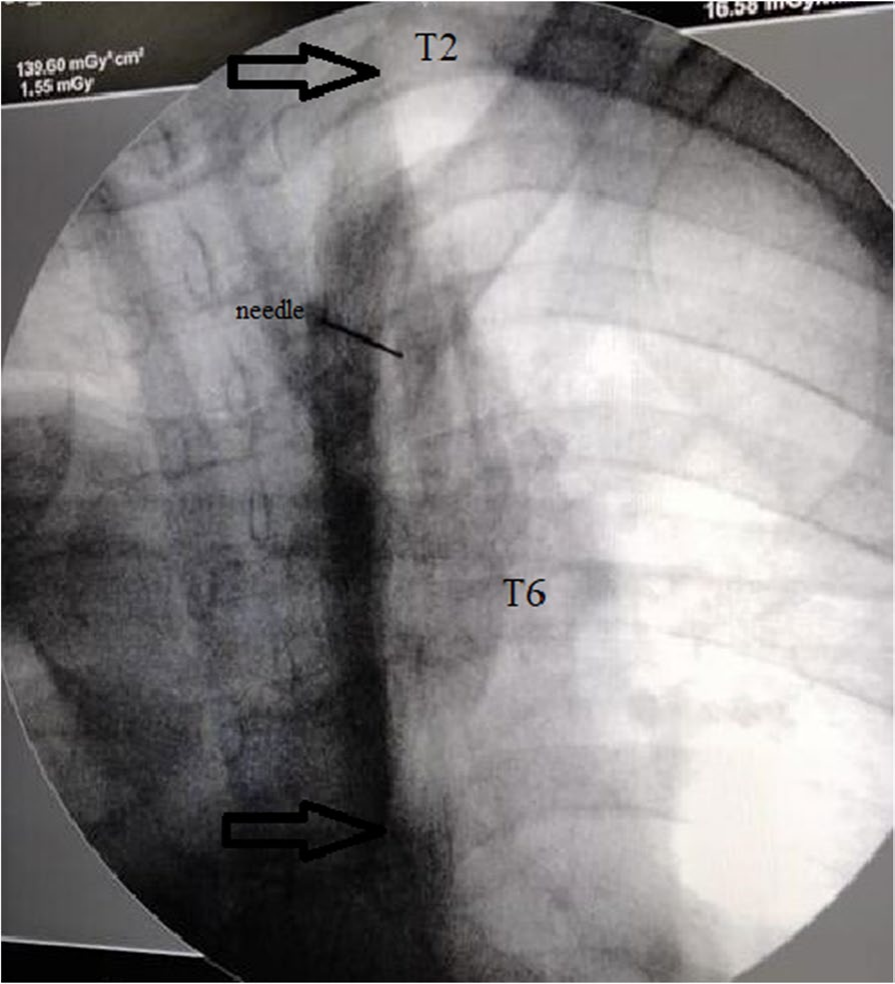
Fluoroscopic photo, arrows show the level of spread in the 20 ml ESB group from the T2 to T6 levels and the needle was at the transverse process of T4

**Fig. 3:**
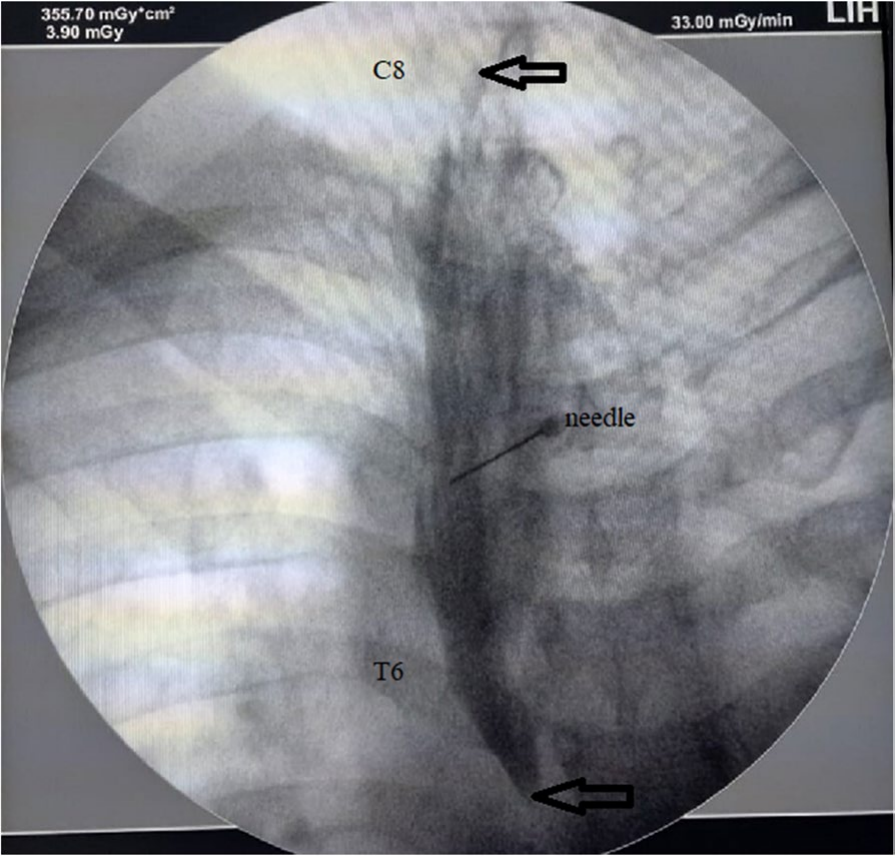
Fluoroscopic photo, arrows show the level of spread in the 30 ml ESB group from the C8 to T6 levels and the needle was at the transverse process of T4

Standard patient monitoring included ECG, non-invasive blood pressure measurement every 5 min, and pulse oximetry. An intravenous cannula was inserted, and resuscitation equipment was kept nearby. The patient was positioned prone. Using fluoroscopic guidance, the transverse process of the T4 vertebra was identified. The target site was sterilized and anesthetized with 3 ml of 2% lidocaine. A 25-gauge spinal needle was inserted to the level of the T4 transverse process. After withdrawing the needle by a distance of 2–3 mm and confirming that it was not intravascular through suction, either 20 ml or 30 ml of the solution was injected. Subsequently, another fluoroscopic antero-posterior photo was taken to determine the number of levels reached by the solution in each group (Figs. 2 and 3). The patient was then transferred to the post-anesthetic care unit and monitored for one hour. Any complications during or after the procedure were documented.

### Study outcomes

The primary outcome was the number of vertebral levels reached by the solution in each volume. Secondary outcomes included the assessment of NRS scores at 1, 2, and 3 months after the injection, patient satisfaction after 3 months (measured on a linear scale ranging from very satisfied to very unsatisfied), the correlation between the levels of solution spread and pain improvement with patient satisfaction, and the occurrence of any complications during or after the injection.

The same anesthesiologist who performed the block assessed the number of vertebral levels reached by the solution and managed any reported complications. NRS scores and patient satisfaction were evaluated by another team member who was unaware of the patients' group assignments.

### Statistical analysis

Data analysis was conducted using the Statistical Package for the Social Sciences (SPSS, version 25). The Shapiro–Wilk test was used to assess data normality. Parametric data were expressed as means ± SD, while non-parametric data were presented as medians and interquartile ranges. Categorical variables were reported as numbers (percentages). One-way ANOVA and Kruskal–Wallis tests were used for continuous data, and the Chi-square test was used for categorical data. Bivariate correlations were assessed using Pearson's or Spearman's correlation coefficients as appropriate. A probability (P) value of ≤ 0.05 was considered statistically significant.

## Results

Sixty-two patients were assessed for eligibility, and 12 patients were excluded, resulting in a study sample of 50 patients (Fig. 1). Table 1 presents the demographic characteristics and ASA classification of the study groups, showing no statistically significant differences (*P* > 0.05).

**Table 1:** Demographic characteristics and ASA classification of the study groups

		The 20 ml group (*n* = 25)	The 30 ml group (*n* = 25)	95% CI
**Age**		44.96 ± 5.697	48.08 ± 5.499	-6.3, 0.1
**Weight**		73.10 ± 10.894	75.47 ± 11.251	-8.7, 3.9
**Height**		1.66 ± 0.039	1.65 ± 0.045	0
**BMI**		26.41 ± 3.71	27.58 ± 3.875	-3.3, 1
**ASA**	**1**	10 (40%)	7 (28%)	-
	**2**	12 (48%)	16 (64%)	
	**3**	3 (12%)	2 (8%)	

Data were expressed as mean ± standard deviation or number (percent)

*ASA* American society of Anesthesiology, *BMI* Body mass index, *95% CI* 95% confidence interval

Table 2 demonstrates significant differences between the study groups in terms of the solution spread in the upper segments (C8, T1, T2, and T3) and lower segments (T5, T6, and T7) with the T4 transverse vertebra as the anatomical landmark (*P* < 0.001 and 0.07, respectively). Additionally, the mean numbers of vertebral blockade were 5.12 ± 0.726 and 6.36 ± 0.569 in the 20 ml and 30 ml groups, respectively (*P* < 0.001). In relation to the C8 to T6 blockade, six patients (24%) were observed in the 30 ml group compared to none in the 20 ml group (*P* = 0.009). Regarding the T1 to T6 blockade, six patients (24%) were found in the 20 ml group compared to 23 patients (92%) in the 30 ml group (*P* < 0.001). Furthermore, 19 patients (76%) and 25 patients (100%) exhibited T2 to T6 blockade in the 20 ml and 30 ml groups, respectively (*P* = 0.009).

**Table 2:** The spread of the injected solution in the study groups

		The 20 ml group (*n* = 25)	The 30 ml group (*n* = 25)	P
**Upper segments**	**C8**	0	6 (24%)	**< 0.001**
	**T1**	7 (28%)	17 (68%)	
	**T2**	16 (64%)	2 (8%)	
	**T3**	2 (8%)	0	
**Lower segments**	**T5**	4 (16%)	0	0.07
	**T6**	19 (76%)	20 (80%)	
	**T7**	2 (8.0%)	5 (20%)	
**Number of blockade levels**	**Mean**	5.12 ± 0.726	6.36 ± 0.569	**< 0.001**
	**4**	5 (20%)	0	**< 0.001**
	**5**	12 (48%)	1 (4%)	
	**6**	8 (32%)	14 (56%)	
	**7**	0	10 (40%)	
**C8 to T6 levels block**		0	6 (24%)	**0.009**
**T1 to T6 levels block**		6 (24%)	23 (92%)	**< 0.001**
**T2 to T6 levels block**		19 (76%)	25 (100%)	**0.009**

Data were expressed as mean ± standard deviation or number (percent)

NRS scores showed improvement in the 30 ml group during the three-month follow-up period compared to the 20 ml group. Within-group comparisons revealed statistically significant differences in NRS scores at 1, 2, and 3 months compared to baseline values. Patient satisfaction was higher in the 30 ml group than the 20 ml group (7.48 ± 1.661 versus 5.04 ± 2.091, *P* < 0.001) (Table 3).

**Table 3:** NRS and patient satisfaction of the study groups

		The 20 ml group (*n* = 25)	The 30 ml group (*n* = 25)	95% CI	P
**NRS**	**Baseline**	7.36 ± 1.469	7.4 ± 1.472	-0.9, 0.8	0.924
	**1 month**	3.88 ± 1.563	2.4 ± 1.528	0.6, 2.4	**0.001**
	**P**_**1**_	**< 0.001**	**< 0.001**		
	**2 months**	4.56 ± 1.502	2.68 ± 1.464	1.0, 2.7	**< 0.001**
	**P**_**1**_	**< 0.001**	**< 0.001**		
	**3 months**	5.68 ± 1.676	3.44 ± 1.583	1.3, 3.2	**< 0.001**
	**P**_**1**_	**< 0.001**	**< 0.001**		
**Patient satisfaction**		5.04 ± 2.091	7.48 ± 1.661	-3.5, -1.4	**< 0.001**

Data were expressed as mean ± standard deviation. P_1_ indicates the difference of each reading compared to the baseline value

*95% CI* 95% confidence interval, *NRS* numerical rating scale

Table 4 presents the correlations between the levels of solution spread and NRS scores with patient satisfaction at three months after the injection. The T1 to T6 blockade demonstrated better NRS scores and patient satisfaction than other blockade levels (*P* < 0.001 and 0.011, respectively).

**Table 4:** Correlations between blockade levels and NRS with patient satisfaction at three months after the injection

	NRS		Patient satisfaction	
	**R**	**P**	**r**	**P**
**Number of blocked levels**	-0.573	**< 0.001**	0.196	0.172
**C8 to T6 levels block**	-0.516	**< 0.001**	0.179	0.214
**T1 to T6 levels block**	-0.690	**< 0.001**	0.356	**0.011**
**T2 to T6 levels block**	-0.430	**0.002**	0.127	0.381

*NRS* numerical rating scale

No complications were reported in either group during the procedure or the follow-up period.

## Discussion

Post-mastectomy pain syndrome (PMPS) is a common problem following breast surgeries, and its prevention and management remain challenging with limited research available [10].

To the best of our knowledge, this study is the first to compare two volumes (20 ml versus 30 ml) of erector spinae block (ESB) in patients with PMPS and assess the levels of dermatomes covered by each volume. We also evaluated the volume that provided the best pain improvement and patient satisfaction in women with post-mastectomy pain.

In our study, we found that the 30 ml solution resulted in a greater number of vertebral blockade levels compared to the 20 ml solution. This interesting finding suggests that the larger volume group may have better pain relief and patient satisfaction. This contributes valuable information to the literature and calls for further large-scale studies to confirm our results.

The mechanism of action of ESB has been investigated radiologically and anatomically on fresh cadavers. Forero et al. found that ESB spreads to the dorsal and ventral rami of the spinal nerves [11]. Diwan et al. examined the spread of a 20 ml radio-contrast dye solution in ESB at the level of the first thoracic costo-transverse junction on two fresh cadavers and observed that the injection affects the dorsal spinal nerves of the cervical and thoracic regions, spreading in the paravertebral space dorsal to the ventral cervical nerve roots [12]. Another study on five cadavers showed that ESB injection at the T4-5 costo-transverse junction spread medially over the retrolaminar space without crossing the midline, and laterally into the paravertebral space, reaching the vertebral foramen, epidural space, and intercostal spaces [13].

Considering the anatomical distribution of thoracic innervation, it is important to note that the areas affected by PMPS receive innervation from different dorsal roots of the spinal nerves. The medial brachial cutaneous nerve arises from C8 and T1 levels and innervates the upper medial aspect of the arm, while the axilla is innervated at the T2 level [14, 15]. The anterior chest wall is innervated from T2 to T6 levels [16]. This explains the better distribution of pain relief with the larger volume solution.

Aoyama et al. compared ESB with 20 ml of 0.5% ropivacaine to paravertebral block for breast surgery and found that the percentage of patients in the paravertebral block group who developed T3 and T5 blockade was higher than in the ESB group [17].

Bang et al. studied the effectiveness of thoracic ESB as analgesia after lung lobectomy and performed the block at the T5 level with a total volume of 30 ml, consisting of a mixture of 15 ml of 0.75% ropivacaine and 15 ml of saline. They observed sensory blockade in the T2-8 dermatomes, with numeric rating scale (NRS) scores of 1 at rest, 3 during active coughing, and 1 during deep breathing [18].

Hasoon et al. reported pain relief for three months in an elderly female with PMPS after ESB at the T5 level using 2 ml of non-ionic contrast, 1 ml of 40 mg/ml of methylprednisolone, and 9 ml of 0.25% bupivacaine [5].

In contrast to our results, Fang et al. stated that ESB with 20 ml of 0.25% bupivacaine alone was insufficient to relieve pain and required additional postoperative sufentanil to achieve effects similar to those of thoracic paravertebral block for patients undergoing thoracotomy. However, they approved that ESB has less frequent side effects [19].

Regarding the reported side effects, Azmy et al. stated that complications of ESB are infrequent because the site of the block is far away from the spinal cord, the pleura, and any major blood vessels [20]. Additionally, Aoyama et al. did not report any serious complications with ESB [17].

### Study limitations

The current research is a single-center study with a relatively small sample size, which could affect the study's power. The sample size is relevant to the level of variability and is likely insufficient to clearly determine if PMPS is reduced in these patients.

## Conclusion

The injection of a 30 ml solution of 0.25% bupivacaine with methylprednisolone in erector spinae block (ESB) may result in better analgesia and higher patient satisfaction in individuals with postmastectomy pain syndrome (PMPS) compared to a 20 ml solution.We recommend conducting large-scale multicenter studies with different validated questionnaires to validate and generalize our findings.

## Data Availability

The datasets used and/or analyzed during the current study are available from the corresponding author upon reasonable request.

## Abbreviations

ASAPS: American Society of Anesthesiology Physical Status
BMI: Body mass index
ESB: Erector spinae block
IRB: Institutional Research Board
NRS: Numerical rating scale
PMPS: Postmastectomy pain syndrome
SPSS: Statistical Package for the Social Sciences
